# Association of Transanal Total Mesorectal Excision With Local Recurrence of Rectal Cancer

**DOI:** 10.1001/jamanetworkopen.2020.36330

**Published:** 2021-02-03

**Authors:** Antonio Caycedo-Marulanda, Lawrence Lee, Sami A. Chadi, Chris P. Verschoor, Jordan Crosina, Shady Ashamalla, Carl J. Brown

**Affiliations:** 1 Department of Surgery, Kingston Health Sciences Centre, Queen’s University, Kingston, Ontario, Canada; 2 Health Sciences North Research Institute, Sudbury, Ontario, Canada; 3 Department of Surgery, McGill University Health Centre, Montreal, Quebec, Canada; 4 Division of Surgical Oncology and General Surgery, University Health Network and Princess Margaret Hospital, University of Toronto, Toronto, Ontario, Canada; 5 Northern Ontario School of Medicine, Sudbury, Ontario, Canada; 6 Department of Surgery Sunnybrook Health Sciences Centre, University of Toronto, Toronto, Ontario, Canada; 7 Department of Surgery, St Paul’s Hospital, The University of British Columbia, Vancouver, British Columbia, Canada

## Abstract

**Importance:**

Proponents of novel transanal total mesorectal excision (TME) suggest the procedure overcomes the technical and oncologic challenges of conventional approaches for treating rectal cancer. Recently, however, there has been controversy regarding the oncologic safety of the procedure.

**Objective:**

To assess the association of transanal TME with the incidence of local recurrence (LR) of cancer and the probability of remaining free of LR during follow-up.

**Design, Setting, and Participants:**

This multicenter cohort study used data from 8 high-volume rectal cancer academic institutions from across Canada on all consecutive patients with primary rectal cancer treated by transanal TME at the participating centers. The study was conducted between January 2014 and December 2018, and data were analyzed from April 1, 2020, to September 15, 2020.

**Exposure:**

Transanal TME.

**Main Outcomes and Measures:**

The incidence of LR was reported as a direct measure of quality of resection. The cumulative probability of LR- and systemic recurrence (SR)–free survival at 36 months was estimated. Local recurrence and SR were defined as radiologic or endoscopic evidence of 1 or more new lesions in or outside the pelvis, respectively, documented during surveillance after the removal of the primary tumor.

**Results:**

Of 608 total patients included in the analysis, 423 (69.6%) were male; the median age was 63 years (interquartile range [IQR], 54-70 years). Local recurrence was identified in 22 patients (3.6%) after a median follow-up of 27 months (IQR, 18-38 months). The median time to LR was 13 months (IQR, 9-19 months). Sixteen of the 22 patients with LR (72.7%) were male, 14 (63.6%) received neoadjuvant chemoradiation, and 12 (54.5%) had American Joint Committee on Cancer stage III disease. Of those with LR, 16 (72.7%) had a negative circumferential radial margin and 20 (9.1%) had a negative distal resection margin, 20 (90.9%) experienced conversion to open surgery, and 15 (68.2%) also developed SR. The probability of LR-free survival at 36 months was 96% (95% CI, 94%-98%). According to the Cox proportional hazards regression model, the hazard ratio of LR was estimated to be 4.2 (95% CI, 2.9-6.2) times higher among patients with a positive circumferential radial margin than among those with a negative circumferential radial margin.

**Conclusions and Relevance:**

In this cohort study, transanal TME performed by experienced surgeons was associated with an incidence of LR and SR that is in line with the published literature on open and laparoscopic TME, suggesting that transanal TME may be an acceptable approach for management of rectal cancer.

## Key Points

**Question:**

Is there an association between transanal total mesorectal excision (TME) for oncologic resection and the incidence of cancer recurrence?

**Findings:**

In this cohort study of 608 patients with rectal cancer who underwent transanal TME, the incidence of local recurrence after 27 months was 3.6%. The cumulative probability of being free of local recurrence at 36 months was 96%.

**Meaning:**

In this study, outcomes associated with transanal TME were comparable with those reported for other approaches, suggesting that the procedure may be a viable option for management of rectal cancer.

## Teaser

This cohort study examines the association between transanal total mesorectal excision for oncologic resection and the incidence of cancer recurrence among patients at 8 academic cancer institutions in Canada.

## Introduction

Transanal total mesorectal excision (TME) combines transanal and abdominal video-assisted techniques to facilitate oncologic rectal resection in a minimally invasive fashion.^1^ Although numerous studies suggest that conventional laparoscopy has oncologic equivalence to open surgery in patients with rectal cancer,^2,3,4,5,6,7^ the technical and ergonomic limitations of operating in the deep pelvis pose a persistent challenge to adoption.^8^ The possible equivalence has been repeatedly shown to be dependent on a high conversion rate to ensure preservation of oncologic principles. Proponents of transanal TME suggest that the rendezvous procedure overcomes these technical and oncologic challenges and avoids conversion.^9,10^ A large number of surgeons globally have adopted transanal TME, with several authors reporting promising short-term results.^11,12,13^ The procedure is technically demanding and difficult to master, and the learning curve is estimated to be between 40 and 50 cases.^14,15,16^

Most of the existing data regarding oncologic safety are based on surrogate pathologic outcomes, such as completeness of the mesorectum and the histopathologic status of the specimen margins.^11,17,18^ Recent studies^16,19,20,21^ with adequate follow-up to analyze local recurrence (LR) suggest that these surrogates may not be adequate to ensure safety of the technique. Similarly, other studies^22,23^ suggest that LR rates were higher in the first 10 procedures of a surgeon’s experience despite the implementation of a structured national training pathway. In Norway, evidence of increased LR with a concerning multifocal pattern in patients who underwent transanal TME led to a nationwide moratorium on the procedure.^24,25^ More recently, the Association of Coloproctology of Great Britain and Ireland halted a national transanal TME adoption initiative and recommended that only select high-volume centers with appropriate training and facilities continue to provide transanal TME until the concerns regarding LR are resolved.^26^ Such findings raise concerns about the widespread implementation and the broad uptake of this novel technique; however, the potential oncologic benefit of a directly visualized distal margin and optimized management of the lower pelvis cannot be ignored.

More data are needed to assess whether using transanal TME to treat patients with rectal cancer is oncologically safe. To this end, we performed a multicenter cohort study to assess the outcomes associated with transanal TME among patients with rectal cancer.

## Methods

### Patient Population

The Canadian taTME Expert Collaboration (CaTaCO), formed in 2019, includes 8 tertiary academic referral centers in Canada.^27^ In this cohort study, all consecutive patients with primary rectal cancer treated by transanal TME at the participating CaTaCO centers between January 2014 and December 2018 had demographic, operative, pathologic, and follow-up data collected and merged into a single database. Procedures performed for benign diagnoses were excluded. This study was individually approved by the research ethics board of each participating institution. The need for informed consent from patients was waived because all personally identifiable information was removed from the data sets. This study followed the Strengthening the Reporting of Observational Studies in Epidemiology (STROBE) reporting guideline.

### Outcomes

The primary outcome was LR. Secondary outcomes included the pathologic quality of the specimen, the incidence of systemic recurrence (SR), disease-free survival, length of stay, overall perioperative morbidity, anastomotic leak, and readmission within 30 days.

Local recurrence was defined as radiologic or endoscopic evidence of 1 or more new pelvic lesions documented during surveillance after the removal of the primary tumor. Systemic recurrence was defined as radiologic evidence of 1 or more new lesions outside the pelvis documented during surveillance after removal of the primary tumor. Disease-free survival was defined as the time to cancer recurrence or death from any cause.^27^ Duration of follow-up was calculated based on the date of resection and the most recent follow-up with the managing team (surgeon, medical or radiation oncologist, or primary physician). Anastomotic leak was defined as evidence of pelvic infection, determined by a combination of symptoms, imaging, and/or radiologic findings.^16^

Complications were reported according to the Clavien-Dindo classification.^28^ Specimens were assessed by dedicated gastrointestinal pathologists, and quality was graded as complete or near complete vs incomplete according to the Nagtegaal classification.^29^ Margins were considered negative if tumor cells were at least 1 mm away from the edge. An intense surveillance program with regular posttreatment assessment of carcinoembryonic antigen values, colonoscopy, and axial imaging was consistent across all centers.^30^

### Procedures

Preoperative investigations included endoscopy; biopsy; carcinoembryonic antigen level; computed tomographic scan of the chest, abdomen, and pelvis; and magnetic resonance imaging (MRI) of the pelvis. Additional or alternative imaging (positron emission tomography–computed tomography, abdominal MRI, and endorectal ultrasonography or repeat pelvic MRI) was requested at the discretion of the managing team. In accordance with Canadian standards, it was encouraged that all cases be presented at a multidisciplinary tumor conference, and candidates for neoadjuvant chemotherapy and/or radiotherapy were treated at a regional cancer center.^30^

Sequential vs simultaneous approaches involving 1 or 2 surgical teams, respectively, as well as the choice of transanal platform depended on the institution and surgeon equipoise. Six centers always used a flexible platform for transanal minimally invasive surgery (GelPOINT path, Applied Medical), 1 center exclusively used a rigid transanal endoscopy microsurgery system (Richard Wolf GmbH), and 1 site had access to both devices. All surgeons had subspecialty training and were involved in hands-on courses with cadaveric models before adopting the technique.

The description of the surgical technique is reported elsewhere.^15,31,32,33^ Most surgeons elected to use the procedure for tumors in the middle and lower third of the rectum, avoiding T4 lesions or those requiring abdominoperineal resections.

### Statistical Analysis

Continuous data are summarized as medians and interquartile ranges (IQRs), and groups were compared by *t* tests or Wilcoxon rank sum tests when applicable; categorical data are summarized as counts and frequencies, and group frequencies were compared by Fisher exact tests. *P* < .05 was considered statistically significant using 2-tailed tests. Associations between the rate of LR or SR and the patient or surgical factors, including body mass index (calculated as weight in kilograms divided by height in meters squared), sex, neoadjuvant therapy, stage, tumor height, quality of the mesorectum, and circumferential and distal margins, were assessed using Cox proportional hazards regression models. These models were clustered by site and had an intraclass correlation of 0.015 (design effect, 2.1) for LR and 0.063 (design effect, 5.7) for SR. Recurrence was regressed on each factor in a univariate manner, and if multiple factors were found to be significantly associated with the outcome, those factors were then entered together into a subsequent multivariable model. The hazard ratio (HR) and 95% CI for LR or SR is reported, along with Kaplan-Meier curves. All analyses were performed using R, version 3.6 (R Project for Statistical Computing). Data were analyzed from April 1, 2020, to September 15, 2020.

## Results

### Patient Characteristics and Surgical Outcomes

A total of 608 patients underwent transanal TME surgery for rectal cancer and were included in the analysis, of whom 423 (69.6%) were male. The median age was 63 years (54-70 years), and the median body mass index was 27.0 (IQR, 24.1-31.3) (Table 1). The caseload for each of the 8 sites where the surgeries were performed was 38, 33, 53, 103, 143, 115, 102, and 21 cases each.

**Table 1.** Patient and Disease Characteristics

Characteristic	Total patients (N = 608)
Age, median (IQR), y	63 (54-70)
Sex, No. (%)	
Female	185 (30.4)
Male	423 (69.6)
Body mass index, No. (%)	
Underweight or normal	188 (30.9)
Overweight	226 (37.2)
Obese	194 (31.9)
Clinical AJCC stage, No. (%)	
I	132 (21.7)
II	166 (27.3)
III	256 (42.1)
IV	35 (5.8)
Missing	19 (3.1)
Tumor height, median (IQR), cm	6 (4-8)
Missing	11 (1.8)
Neoadjuvant therapy, No. (%)	
No	183 (30.1)
Yes	425 (69.9)

Abbreviations: AJCC, American Joint Committee on Cancer; IQR, interquartile range.

The median tumor height was 6 cm (IQR, 4-8 cm), and 256 patients (42.1%) had clinical stage III disease, followed by 166 (27.3%) with stage II disease, 132 (21.7%) with stage I disease, and 35 (5.8%) with stage IV disease. Neoadjuvant chemotherapy and/or radiotherapy was delivered to 425 patients (69.9%). A low anterior resection was performed for 559 patients (91.9%), whereas 44 (7.2%) underwent an abdominoperineal resection and 3 (0.5%) underwent another form of resection (Table 2). Most patients (371 [61.0%]) had a stapled anastomosis, whereas 178 (29.3%) had a handsewn reconstruction and 52 (8.6%) had no anastomosis. Of those who underwent reconstruction, 525 (86.3%) received a diverting ileostomy. The median operative time was 276 minutes (IQR, 235-338 minutes), the conversion rate was 4.3% (26 patients), and the intraoperative complication rate was 4.9% (30 patients).

**Table 2.** Complete Stratified Outcomes by Patient and Surgical Characteristics^a^

Characteristic	Total sample (N = 608)	Local recurrence		Systemic recurrence^b^	
		No (n = 586)	Yes (n = 22)	No (n = 526)	Yes (n = 47)
Age, median (IQR), y	63 (54-70)	63 (54-70)	67 (47-74)	63 (54-70)	67 (52-72)
Sex					
Female	185 (30.4)	179 (30.6)	6 (27.3)	156 (29.7)	14 (29.8)
Male	423 (69.6)	406 (69.4)	16 (72.7)	370 (70.3)	33 (70.2)
Body mass index					
Underweight or normal	188 (30.9)	179 (30.6)	9 (40.9)	156 (29.7)	16 (34.0)
Overweight	226 (37.2)	215 (36.8)	10 (45.5)	194 (36.9)	21 (44.7)
Obese	194 (31.9)	191 (32.6)	3 (13.6)	176 (33.5)	10 (21.3)
Clinical AJCC stage					
I	132 (21.7)	127 (21.7)	5 (22.7)	126 (24.0)	6 (12.8)
II	166 (27.3)	163 (27.9)	3 (13.6)	155 (29.5)	11 (23.4)
III	256 (42.1)	243 (41.5)	12 (54.5)	226 (43.0)	30 (63.8)
IV	35 (5.8)	33 (5.6)	2 (9.1)	0	0
Missing	19 (3.1)	19 (3.2)	0	19 (3.6)	0
Tumor height					
Median (IQR), cm	6 (4-8)	6 (4-8)	5 (3-7)	6 (4-8)	6 (5-8)
Missing	11 (1.8)	11 (1.9)	0	9 (1.7)	0
Neoadjuvant therapy					
No	183 (30.1)	175 (29.9)	8 (36.4)	170 (32.3)	9 (19.1)
Yes	425 (69.9)	410 (70.1)	14 (63.6)	356 (67.7)	38 (80.9)
Procedure					
APR resection	44 (7.2)	41 (7.0)	3 (13.6)	40 (7.6)	4 (8.5)
LAR	559 (91.9)	539 (92.1)	19 (86.4)	481 (91.4)	43 (91.5)
Other	3 (0.5)	3 (0.5)	0	3 (0.6)	0
Missing	2 (0.3)	2 (0.3)	0	2 (0.4)	0
Anastomosis type					
Handsewn	178 (29.3)	168 (28.7)	10 (45.5)	157 (29.8)	15 (31.9)
None	52 (8.6)	49 (8.4)	3 (13.6)	44 (8.4)	6 (12.8)
Stapled	371 (61.0)	362 (61.9)	8 (36.4)	319 (60.6)	26 (55.3)
Missing	7 (1.2)	6 (1.0)	1 (4.5)	6 (1.1)	0
Use of diverting ostomy					
Any stoma	525 (86.3)	507 (86.7)	17 (77.3)	451 (85.7)	43 (91.5)
No stoma	71 (11.7)	67 (11.5)	4 (18.2)	63 (12.0)	4 (8.5)
Missing	12 (2.0)	11 (1.9)	1 (4.5)	12 (2.3)	0
Operative time					
Median (IQR), min	276 (235-338)	276 (234-338)	300 (249-366)	275 (237-338)	290 (246-332)
Missing	3 (0.5)	3 (0.5)	0	2 (0.4)	0
Conversion					
No	581 (95.6)	560 (95.7)	20 (90.9)	505 (96.0)	44 (93.6)
Yes	26 (4.3)	24 (4.1)	2 (9.1)	21 (4.0)	3 (6.4)
Missing	1 (0.2)	1 (0.2)	0	0	0
Intraoperative complications					
No	575 (94.6)	552 (94.4)	22 (100)	495 (94.1)	46 (97.9)
Yes	30 (4.9)	30 (5.1)	0	29 (5.5)	0
Missing	3 (0.5)	3 (0.5)	0	2 (0.4)	1 (2.1)
Postoperative complications					
No	269 (44.2)	262 (44.8)	7 (31.8)	228 (43.3)	21 (44.7)
Yes	339 (55.8)	323 (55.2)	15 (68.2)	298 (56.7)	26 (55.3)
Clavien-Dindo classification					
1	108 (17.8)	102 (17.4)	5 (22.7)	99 (18.8)	5 (10.6)
2	135 (22.2)	131 (22.4)	4 (18.2)	117 (22.2)	11 (23.4)
3	88 (14.5)	83 (14.2)	5 (22.7)	75 (14.3)	9 (19.1)
4	5 (0.8)	4 (0.7)	1 (4.5)	4 (0.8)	1 (2.1)
5	3 (0.5)	3 (0.5)	0	3 (0.6)	0
Anastomotic leak					
No	559 (91.9)	541 (92.5)	17 (77.3)	484 (92.0)	42 (89.4)
Yes	46 (7.6)	41 (7.0)	5 (22.7)	39 (7.4)	5 (10.6)
Missing	3 (0.5)	3 (0.5)	0	3 (0.6)	0
Another operation within 30 d					
No	544 (89.5)	524 (89.6)	19 (86.4)	470 (89.4)	43 (91.5)
Yes	64 (10.5)	61 (10.4)	3 (13.6)	56 (10.6)	4 (8.5)
Readmission within 30 d					
No	508 (83.6)	490 (83.8)	17 (77.3)	444 (84.4)	36 (76.6)
Yes	100 (16.4)	95 (16.2)	5 (22.7)	82 (15.6)	11 (23.4)
Quality of mesorectum					
Incomplete	27 (4.4)	26 (4.4)	1 (4.5)	23 (4.4)	4 (8.5)
Complete	565 (92.9)	546 (93.3)	18 (81.8)	490 (93.2)	40 (85.1)
Missing	16 (2.6)	13 (2.2)	3 (13.6)	13 (2.5)	3 (6.4)
Circumferential radial margin					
Positive	43 (7.1)	38 (6.5)	4 (18.2)	31 (5.9)^c^	10 (21.3)^c^
Negative	505 (83.1)	489 (83.6)	16 (72.7)	441 (83.8)^c^	35 (74.5)^c^
Missing	60 (9.9)	58 (9.9)	2 (9.1)	54 (10.3)^c^	2 (4.3)^c^
Distal radial margin					
Positive	15 (2.5)	15 (2.6)	0	10 (1.9)^d^	4 (8.5)^d^
Negative	533 (87.7)	513 (87.7)	20 (90.9)	463 (88.0)^d^	41 (87.2)^d^
Missing	60 (9.9)	57 (9.7)	2 (9.1)	53 (10.1)^d^	2 (4.3)^d^
Local recurrence					
No	585 (96.2)	585 (100)^e^	0^e^	518 (98.5)^e^	34 (72.3)^e^
Yes	22 (3.6)	0^e^	22 (100)^e^	7 (1.3)^e^	13 (27.7)^e^
Missing	1 (0.2)	0^e^	0^e^	1 (0.2)^e^	0^e^
Systemic recurrence					
No	551 (90.6)	543 (92.8)^e^	7 (31.8)^e^	526 (100)^e^	0^e^
Yes	57 (9.4)	42 (7.2)^e^	15 (68.2)^e^	0^e^	47 (100)^e^
Follow-up, median (IQR), mo	27 (18-38)	27 (18-38)	36 (22-40)	27 (18-38)	34 (23-42)^d^

Abbreviations: AJCC, American Joint Committee on Cancer; APR, abdominoperineal; IQR, interquartile range; LAR, low anterior resection.

^a^ Data are presented as number (percentage) of patients unless otherwise indicated.

^b^ Stage IV cases (35) were not considered in the stratified analysis of systemic recurrence.

^c^ *P* = .001.

^d^ *P* < .05.

^e^ *P* < .001.

Postoperative complications were observed in 339 patients (55.8%); grade III Clavien-Dindo complications occurred in 88 patients (14.5%) and grade IV in 5 patients (0.8%) (Table 2). Complications that appeared to be associated with the transanal portion included 2 urethral injuries, 1 vaginal injury, and 3 instances of defects made in the rectum. Of the 3 patients who had a defect made in the rectal wall, 1 subsequently experienced LR. There were 6 episodes of presacral bleeding, although the data did not capture whether these occurred during the laparoscopic or the transanal portion of the dissection.

The anastomotic leak rate within the initial 30 postoperative days was 7.6% (46 patients), and readmission was required for 100 (16.4%) patients. Four patients died within 30 days of surgery during the study period. One experienced a fatal stroke on postoperative day 4; another had an anastomotic leak and abscess formation and ultimately died on postoperative day 17. One patient had an uncomplicated course in the immediate postoperative period and was discharged home on postoperative day 7; this patient died of unknown causes on postoperative day 28. On postoperative day 17, the other patient was presumed to have a perforated viscus based on an acute presentation with findings of free air; this patient experienced rapid decompensation, received palliative treatment, and died.

### Oncologic Outcomes

**Figure 1.**
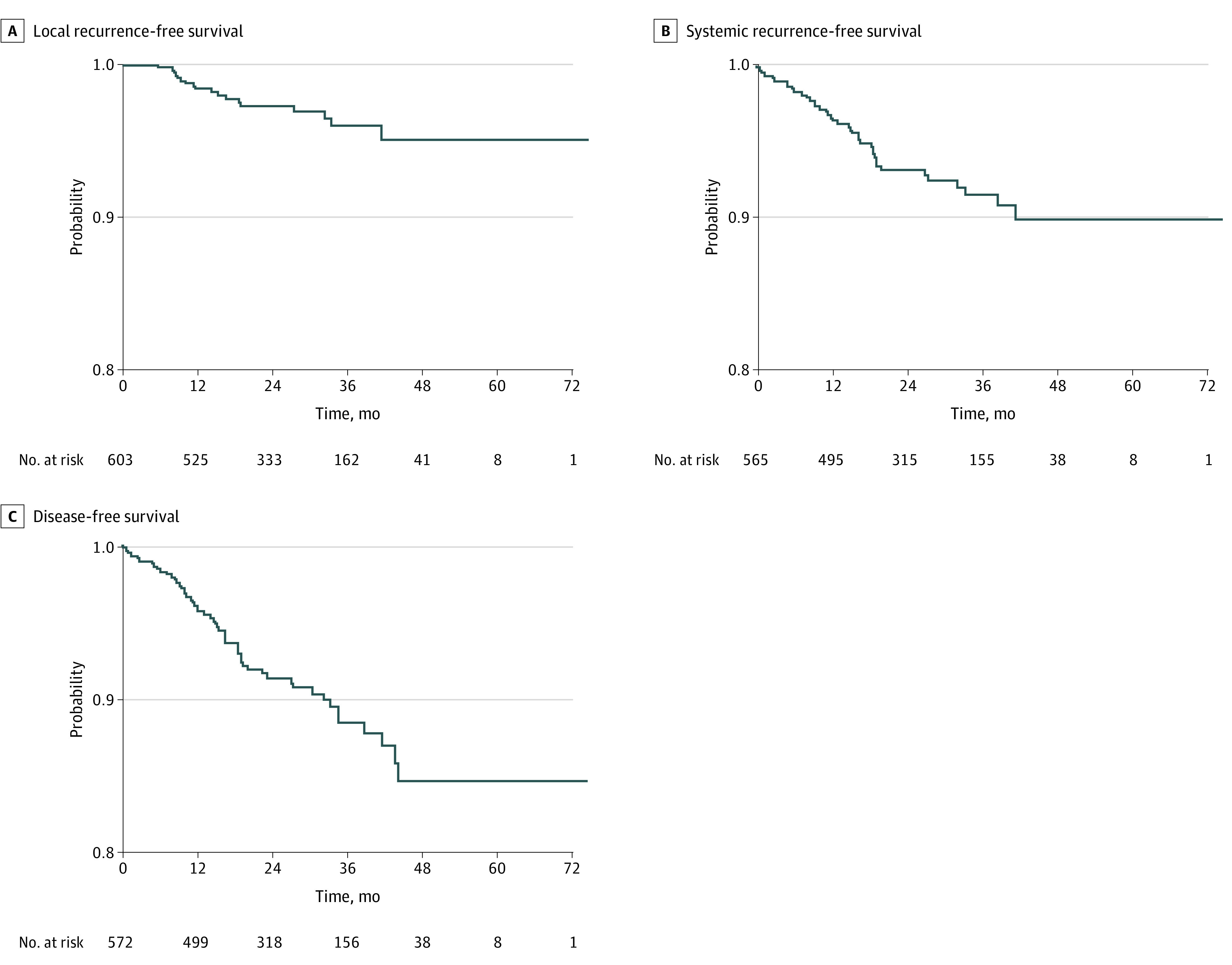
Kaplan-Meier Curves Presenting the Probability of Local Recurrence-Free, Systemic Recurrence-Free, and Disease-Free Survival During the Course of the Study

The rate of LR was 3.6% (22 patients) after a median follow-up of 27 months (IQR 18-38 months), and the median time to LR was 13 months (IQR, 9-19 months) (Table 2). Of the 22 patients with LR, 16 (72.7%) were male, 14 (63.6%) received neoadjuvant chemoradiation, and 12 (54.5%) had American Joint Committee on Cancer stage III disease. Of those with LR, 16 (72.7%) had a negative circumferential radial margin (CRM), and 20 (90.9%) had a negative distal resection margin (DRM); 2 (9.1%) experienced conversion to open surgery; and 15 (68.2%) also developed SR. Three patients from different centers among the first 10 cases developed LR. The probability of LR-free survival at 24 months was 97% (95% CI, 95%-99%), and at 36 months it was 96% (95% CI, 94%-98%) (Figure 1). The probability was reduced among patients with a positive CRM (90%; 95% CI, 81%-100%) compared with those with a negative CRM (96%; 95% CI, 93%-98%) (eFigure in the Supplement) and did not differ according to DRM status or disease stage. According to the Cox proportional hazards regression model, the hazard of LR was estimated to be 4.19 (95% CI, 2.86-6.15) times higher among patients with a positive CRM compared with those with a negative CRM (Table 3).

**Table 3.** Cox Proportional Hazards Regression Analysis of the Association of Local and Systemic Recurrence With Patient and Surgical Factors^a^

Characteristic	Hazard ratio (95% CI)		
	Local recurrence, univariate	Systemic recurrence	
		Univariate	Multivariate
Body mass index			
Underweight or normal	1 [Reference]	1 [Reference]	NA
Overweight	0.96 (0.38-2.45)	0.99 (0.48-2.04)	NA
Obese	0.41 (0.09-1.86)	0.59 (0.33-1.05)	NA
Sex			
Female	1 [Reference]	1 [Reference]	NA
Male	1.62 (0.85-3.07)	0.94 (0.59-1.47)	NA
Neoadjuvant therapy			
No	1 [Reference]	1 [Reference]	1 [Reference]
Yes	0.75 (0.31-1.83)	1.99 (1.27-3.13)^b^	1.70 (0.90-3.21)
AJCC stage (clinical)			
I	1 [Reference]	1 [Reference]	1 [Reference]
II	0.60 (0.36-1.01)	1.35 (0.73-2.49)	0.72 (0.34-1.55)
III	1.42 (0.83-2.44)	2.30 (1.31-4.03)^b^	1.17 (0.46-2.98)
IV	1.26 (0.14-10.99)	NA^c^	NA
Tumor height	0.92 (0.76-1.12)	1.03 (0.93-1.14)	NA
Quality of mesorectum			
Incomplete	1 [Reference]	1 [Reference]	NA
Complete	0.63 (0.08-5.06)	0.49 (0.13-1.76)	NA
CRM			
Negative	1 [Reference]	1 [Reference]	1 [Reference]
Positive	4.19 (2.86-6.15)^b^	4.00 (2.22-7.19)^b^	2.95 (1.26-6.91)^b^
DRM			
Negative	1 [Reference]	1 [Reference]	1 [Reference]
Positive	NA^d^	4.88 (1.55-15.33)	3.37 (0.72-15.74)

Abbreviations: AJCC, American Joint Committee on Cancer; CRM, circumferential radial margin; DRM, distal resection margin; NA, not applicable.

^a^ Both models were clustered by site, and the multivariable model was adjusted for neoadjuvant therapy, stage, CRM, and DRM.

^b^ Significant association.

^c^ Patients with disease stage IV were removed before data were analyzed.

^d^ A reliable estimate could not be derived owing to a lack of cases.

**Figure 2.**
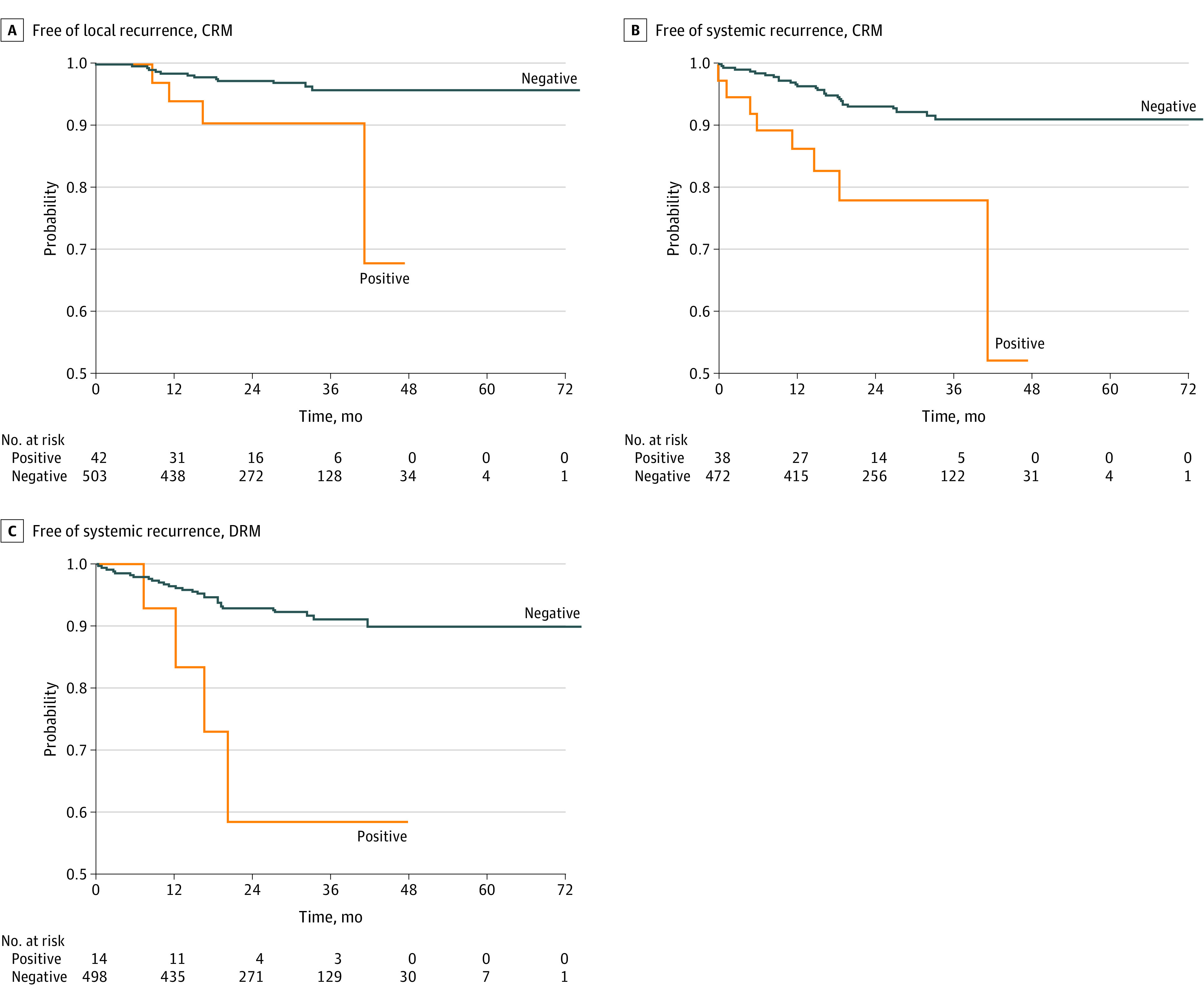
Kaplan-Meier Curves Presenting the Probability of Being Free of Local Recurrence or Systemic Recurrence During the Course of the Study, Stratified by Circumferential Radial Margin (CRM) or Distal Resection Margin (DRM) Status

During the follow-up period, a total of 57 patients (9.4%) developed SR, 47 of whom did not have stage IV disease (Table 2); the probability of being SR free at 36 months in this subset was 92% (95% CI, 89%-94%) (Figure 1). The probability was reduced among patients with a positive CRM (78%; 95% CI, 65%-95%) compared with those with a negative CRM (91%; 95% CI, 88%-95%) (Figure 2), similar to the reduction observed among patients with a positive DRM (59%; 95% CI, 33%-100%) compared with patients with a negative DRM (91%; 95% CI, 88%-94%) (Figure 2). The probability of being SR free at 36 months was also lower among patients with disease stage II (93%; 95% CI, 89%-98%) or III (88%; 95% CI, 83%-93%) than among patients with stage I disease (95%; 95% CI, 90%-99%) (eFigure 3 in the Supplement). After removing the 35 patients with initial stage IV disease from the data analysis, the rate of SR was significantly higher among patients with stage III disease than among those with stage I disease (HR, 2.30; 95% CI, 1.31-4.03), among patients with a positive CRM than among those with a negative CRM (HR, 4.00; 95% CI, 2.22-7.19), and among patients who received neoadjuvant therapy than among those who did not receive therapy (HR, 1.99; 95% CI, 1.27-3.13). In a multivariable model including disease stage, neoadjuvant therapy, CRM, and DRM, only a positive CRM remained significantly associated with SR (adjusted HR, 2.95; 95% CI, 1.26-6.91).

The probabilities of disease-free survival at 24, 36, and 48 months were 91% (95% CI, 89%-94%), 88% (95% CI, 85%-92%), and 85% (95% CI, 80%-90%), respectively (eFigure in the Supplement). Disease-free survival for individual disease stages is shown in eTable 1 in the Supplement.

## Discussion

To our knowledge, the current study is the largest to report on surgical and midterm oncologic outcomes associated with transanal TME from a single country. This cohort study reports the short- and medium-term oncologic outcomes for 608 patients treated with the transanal TME technique for rectal cancer at 8 high-volume academic Canadian institutions during a 5-year period. All of the participating centers were also part of CaTaCO, the intent of which was to develop recommendations and guidelines of the appropriate indications and implementation for this technique in Canada. In this study, the probabilities for 2- and 3-year LR-free survival associated with transanal TME were 97% and 96%, respectively.

The LR rates observed in the present study are consistent with those presented in the Colorectal Cancer Laparoscopic or Open Resection II (COLOR II),^34^ American College of Surgeons Oncology Group Z6051,^35^ and Australasian Laparoscopic Cancer of the Rectum Trial (ALaCaRT)^36^ trials as well as a recent multicenter transanal TME cohort study.^21^ In these publications, 3-year LR rates were reported to be approximately 5% in the laparoscopic TME arm, with minimal to no differences in the open surgery arm. Furthermore, other factors found to be associated with outcomes, such as mesorectal excision grade, CRM involvement, and neoadjuvant chemotherapy and/or radiotherapy, were consistent between this study and the reported literature. These results suggest that high-quality surgical outcomes and acceptable oncologic outcomes are associated with performance of transanal TME by an expert. Although the positive CRM rate in this study was higher than that reported in the recent international transanal TME registry study (4.0%),^37^ the CaTaCO participating hospitals may have used transanal TME for patients with more advanced tumors, which would be expected for high-volume, specialized institutions. Beyond the risk associated with margin positivity, it has been suggested that LR after transanal TME may be associated with technical factors such as the shedding of tumor cells before closing the rectal lumen or failure of the purse-string suture to contain the intraluminal tumor. In this study, the hazard of LR or SR increased more than 3-fold among patients with positive CRM and remained significant for SR even after adjusting for additional patient and procedural factors.

Transanal TME has received increased scrutiny regarding its safety and oncologic effectiveness. In the publications from Norway,^24,25^ where a moratorium was placed on transanal TME, local recurrence was 7.6% among 157 transanal TME cases, with a cumulative 2.4-year local recurrence rate of 11.6%. However, closer examination of the data suggests that many of the factors associated with local recurrence in rectal cancer were either not reported, excluded from the analysis, or not consistent with the generally accepted rates in the literature. These missing data make it difficult to generalize the results. Despite a centralization of rectal cancer surgery and transanal TME to a few specialized centers, the 4 hospitals with the highest volume in the Norwegian studies performed a maximum of 57 cases. Despite their high-volume status, these hospitals reported a 13% incidence of R1 resection, of which 5% had CRM involvement. These findings suggest that the remainder of R1 cases, although not explicitly reported, involved the distal margin and therefore may be reflective of technical failure. Furthermore, the proportion of patients who received neoadjuvant chemoradiation was lower than in other transanal TME series (and rectal cancer series in general)^20,21,22^; only 1 of 12 patients with local recurrences received neoadjuvant chemoradiation despite there being a pathologic T stage of pT3 or above in 10 of 12 patients. Although the Association of Coloproctology of Great Britain and Ireland subsequently released its own cautionary recommendations^25^ after the publication of the Norwegian studies,^23,24^ it is unclear whether the aforementioned data reflect technical inadequacies of a series of early cases or constitute a real indictment of the technique given the inherent limitations.

The CaTaCO has recently published guidelines for the safe use of transanal TME, focusing on institution, surgeon, patient selection, and quality assurance.^27^ These guidelines recommend that surgeons and hospitals only perform transanal TME if they initially have an adequate case volume, multidisciplinary expert support, and extensive experience in both minimally invasive rectal cancer surgery and transanal endoscopic surgery. At present, national credentialing and quality assurance oversight do not exist in Canada. Therefore, implementation and regulation of transanal TME is left to individual surgeons and their institutions. These data represent the combined experience of 8 academic Canadian centers.

The implementation phase of the learning curve may affect oncologic outcomes because LR has been reported to be higher in the first 10 cases performed using the transanal TME technique.^22,23^ In the current study, 3 of 22 cases of LR (13.6%) occurred in patients who underwent transanal TME within the initial 10 cases per center; none of these patients had positive margins or incomplete specimens.

Adverse outcomes in this study cohort did not cluster particularly to the earliest cases: 2 of 4 patients who died within 30 days of surgery were among the first 10 cases performed at their respective centers, and the first 10 cases per center also accounted for 6 of 30 reported intraoperative complications. Of the 2 urethral injuries reported in this cohort, 1 occurred among the first 10 cases. Two presacral bleeds and 2 positive air leak tests requiring suture reinforcement were also seen in the first 10 cases. Two of the 4 deaths occurred among the first 10 cases seen at a center. The 2 deaths among the first 10 cases included 1 patient who experienced a fatal stroke on postoperative day 4 and another who experienced an anastomotic leak and abscess formation and ultimately died on postoperative day 17.

### Limitations

This study has limitations. Because this was a multicenter retrospective cohort sample, there were no defined patient selection criteria and there was no technique standardization. Therefore, there may have been different indications for transanal TME among institutions. Some centers used transanal TME liberally, whereas others reserved it for cases anticipated to be more difficult. There was also no clear documentation of the degree to which TME dissection was conducted transanally; transanal TME was used to dissect as far proximally as possible at 1 center, whereas for other centers, laparoscopic TME conversion to transanal TME depended on the difficulty to optimize the distal margin from the abdominal approach. Further heterogeneity between the type of neoadjuvant therapy protocols and interval to surgery may have affected the difficulty of the surgery as well as the oncologic outcome.^38,39,40^ We did not homogeneously collect data on CRM status on MRI, and we also did not have data from other TME approaches to compare current results; thus, whether transanal TME represents the most appropriate approach in this population is unknown. In addition, although all patients in the study had some form of follow-up, the length of follow-up was relatively short; however, the majority of recurrences occur within the first 12 to 24 months,^21,23,25,34,41^ suggesting that most of the LR would have been captured within this period.

## Conclusions

In this cohort study, transanal TME performed by experienced surgeons was associated with an incidence of LR and SR that is in line with the published literature on open and laparoscopic TME, suggesting that transanal TME may be an acceptable approach for management of rectal cancer. A few ongoing randomized clinical trials are expected to eventually provide definitive evidence regarding the safety profile of transanal TME.

## Notes

Supplement. **eFigure.** Kaplan-Meier curves presenting the probability of being I) local and II) systemic recurrence-free over the course of the study, stratified by disease stage **eTable.** Disease free survival (DFS) probability by stage at 24, 36, 48 months
