# Errors in the Results Section of the Abstract

**DOI:** 10.1001/jamanetworkopen.2021.3659

**Published:** 2021-03-04

In the Original Investigation titled “Association of Transanal Total Mesorectal Excision With Local Recurrence of Rectal Cancer,”^1^ published February 3, 2021, in the Results section of the Abstract, the percentage of patients with local recurrence who had a negative distal resection margin was changed from 9.1% to 90.9% and the number (percentage) of patients with local recurrence who experienced conversion to open surgery was changed from 20 (90.9%) to 2 (9.1%). This article has been corrected.^1^
