# Supplementary material for: Association of Transanal Total Mesorectal Excision With Local Recurrence of Rectal Cancer
Source: JAMA Netw Open. 2021 Feb 3;4(2):e2036330. doi: 10.1001/jamanetworkopen.2020.36330 (PMC7859847; doi:10.1001/jamanetworkopen.2020.36330)
Supplement: Supplement. — eFigure. Kaplan-Meier curves presenting the probability of being I) local and II) systemic recurrence-free over the course of the study, stratified by disease stage eTable. Disease free survival (DFS) probability by stage at 24, 36, 48 months [file jamanetwopen-e2036330-s001.pdf]

Supplementary Online Content

Caycedo-Marulanda A, Lee L, Chadi SA, et al; Canadian taTME Expert Collaboration. Association of transanal total mesorectal excision with local recurrence of rectal cancer. *JAMA Netw Open*. 2021;4(2):e2036330. doi:10.1001/jamanetworkopen.2020.36330

eFigure. Kaplan-Meier curves presenting the probability of being I) local and II) systemic recurrence-free over the course of the study, stratified by disease stage
eTable. Disease free survival (DFS) probability by stage at 24, 36, 48 months

This supplementary material has been provided by the authors to give readers additional information about their work.

© 2021 Caycedo-Marulanda et al. *JAMA Network Open*.

eTable

Stage(Clinical)	Time	DFS probability %
I	24	93.8 [89.4, 98.4]
	36	93.8 [89.4, 98.4]
	48	89.5 [80.7, 99.2]
II	24	95.2 [91.8, 98.8]
	36	92.8 [88.1, 97.7]
	48	87.7 [79.8, 96.4]
III	24	86.8 [82.4, 91.5]
	36	81.5 [75.4, 88]
	48	78.6[70.9,87.2]

Disease Free Survival (DFS) probability by stage at 24,36,48 months

© 2021 Caycedo-Marulanda et al. *JAMA Network Open*.

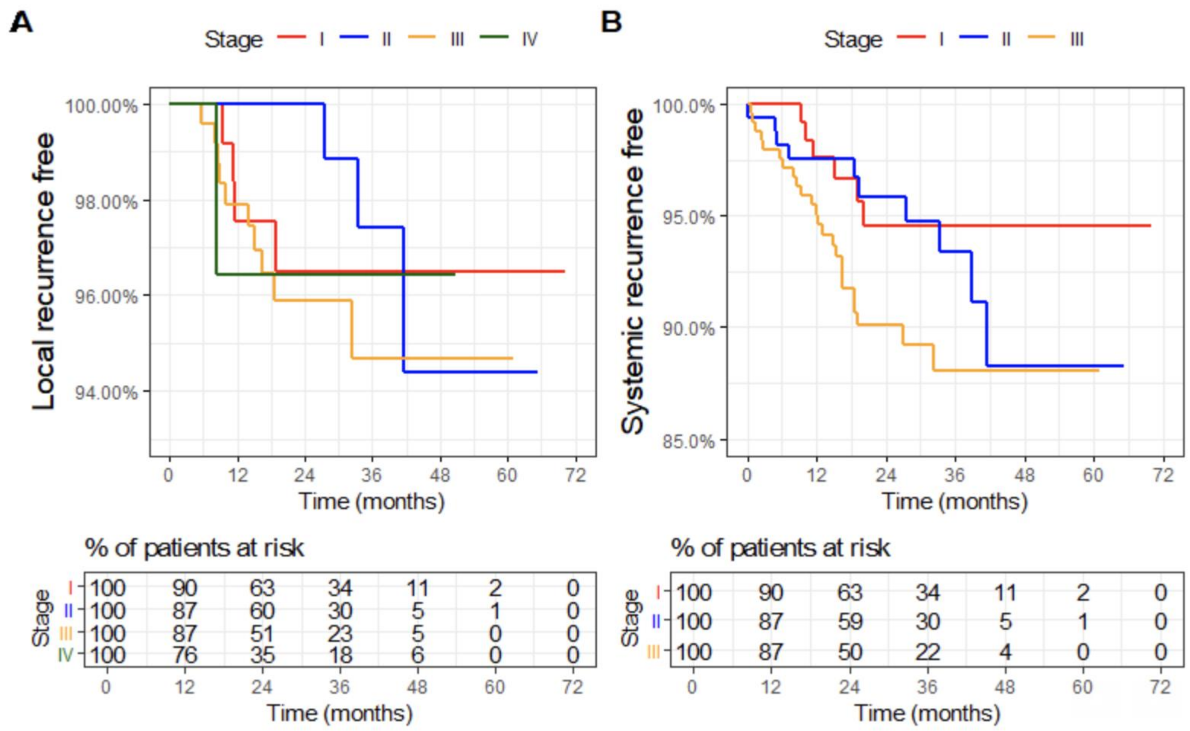

eFigure : *Kaplan-Meier curves presenting the probability of being I) local and II) systemic recurrence-free over the course of the study, stratified by disease stage; censored patients are indicated by vertical tick marks. Note, patients with disease stage IV were removed prior to the analysis of systemic recurrence.*

© 2021 Caycedo-Marulanda et al. *JAMA Network Open*.
